# A Soft Exoskeleton Glove for Hand Bilateral Training via Surface EMG

**DOI:** 10.3390/s21020578

**Published:** 2021-01-15

**Authors:** Yumiao Chen, Zhongliang Yang, Yangliang Wen

**Affiliations:** 1 School of Art, Design and Media, East China University of Science and Technology, Shanghai 200237, China; 2 College of Mechanical Engineering, Donghua University, Shanghai 201620, China; yzl@dhu.edu.cn (Z.Y.); wenwen202012@126.com (Y.W.)

**Keywords:** exoskeleton, surface electromyography, hand motion recognition, bilateral training

## Abstract

Traditional rigid exoskeletons can be challenging to the comfort of wearers and can have large pressure, which can even alter natural hand motion patterns. In this paper, we propose a low-cost soft exoskeleton glove (SExoG) system driven by surface electromyography (sEMG) signals from non-paretic hand for bilateral training. A customization method of geometrical parameters of soft actuators was presented, and their structure was redesigned. Then, the corresponding pressure values of air-pump to generate different angles of actuators were determined to support four hand motions (extension, rest, spherical grip, and fist). A two-step hybrid model combining the neural network and the state exclusion algorithm was proposed to recognize four hand motions via sEMG signals from the healthy limb. Four subjects were recruited to participate in the experiments. The experimental results show that the pressure values for the four hand motions were about −2, 0, 40, and 70 KPa, and the hybrid model can yield a mean accuracy of 98.7% across four hand motions. It can be concluded that the novel SExoG system can mirror the hand motions of non-paretic hand with good performance.

## 1. Introduction

As a cardiovascular disease, stroke is common in the elderly. It often occurs with paralysis, which inhibits the motor ability of the patients. Especially, loss of the ability to move the hands and fingers can reduce one’s quality of daily life considerably [1]. It has been demonstrated that rehabilitation training using the specified assistant systems can help regain function in the affected limb [2]. Most stroke patients have a strong demand for follow-up continuous care at home. However, professional rehabilitation therapy often costs a lot and is not suitable for ordinary patients [3,4]. Therefore, there is an urgent need for a system that can make hand rehabilitation more accessible and comfortable.

Recently, there are many research groups that are developing exoskeletons and wearable robots for hand rehabilitation [5,6]. At present, rigid robot technology has been relatively mature and widely used [7]. However, these rigid exoskeletons still have some limitations, such as being cumbersome, inconvenient operation, limited freedom, difficult structural design, complex control system, and poor portability due to excessive weight [7,8]. Rigid exoskeletons use actuators that are less compliant than the joints themselves [4], so they cannot provide users with good wearable comfort [9]. In contrast, the environmental adaptability and mechanical compliance of soft exoskeletons are better, so they can provide safer human–robotic interactions [10]. Exoskeletons made of lightweight and low-profile textiles are more comfortable and cheaper than those of hard-mechanical structure [11]. One of the earliest soft exoskeletons for hand rehabilitation was a pneumatic glove with polyurethane air bladders [12]. A personal computer with a Visual Basic program was used to control the glove to practice grasp-and-release motions. Therefore, soft exoskeletons have greater market prospects than rigid ones [13,14].

The exoskeletons can be controlled by programming, voice command, and surface electromyography (sEMG) [15]. However, it is difficult for programming control to realize the flexible jump of action. Voice command control can involve patients’ decisions into rehabilitation training naturally. However, voice control systems are susceptible to environmental noise, and do not allow fast feedback or error correction [15]. By contrast, the sEMG-based control can provide continuously variable or fast feedback commands [15]. As a non-invasive and painless measure of the electrical potential present on the skin from a muscle contraction, sEMG has been successfully applied in hand motion recognition for exoskeletons and prostheses. For example, Vogel et al. [16] proposed a robotic arm DLR LWR-III based on sEMG remote control. There have been several scientific efforts aiming to apply machine learning algorithms, such as neural network [17], SVM [16], random forest [18], and so on, to investigate the sEMG-driven exoskeletons. 

Unilateral training and bilateral training are two main strategies for exoskeleton control. Unilateral training is performed on the impaired hand, and the motion intention is directly detected from the impaired hand. Meeker et al. [18] explored a glove with exotendon network, which can detect the user’s intention from sEMG signals to control hand to open or close. Bilateral training can assist the exercise of the impaired limb with the help of the healthy limb. Stoykov et al. [19] illustrated that the effect of bilateral training was significantly higher than that of unilateral training. The reason for this is that patients could express and implement the intention of movement during bilateral training autonomously. Leonardis et al. [17] proposed a hand exoskeleton, BRAVO, that could perform on the paretic hand to assist grasp. It was made of metal, driven by motors, and its total weight was about 950 g. The guidance grasping force for the BRAVO was estimated via sEMG activities registered from the intact hand.

Most recently, many researchers have focused on the design of actuators for soft hand exoskeletons. For example, Whitesides’s team [20] proposed a soft grasp robot based on the PneuNet. On this basis, Polygerinos et al. [4] developed an elastomeric actuator. Besides, they designed an elastomer actuator with fiber reinforcements that could induce specific bending, twisting, and extending trajectories under fluid pressurization [21]. Yap’s team fabricated a glove with bending actuators that were made of two different types of silicone rubber [22]. They optimized the design of the gloves through fabric regulating to constrain radial expansion [23]. Variable stiffness for the actuators was introduced to conform to the finger profile [24]. Also, inflatable plastic actuators were set to provide torques for finger extension [25].

The option to integrate the above mentioned sEMG-based control method and bilateral training into soft exoskeletons are currently being investigated by an increasing number of research groups. Polygerinos’s team constructed a rehabilitation assistant system with the glove that could detect user’s intent of hand closing or opening by monitoring forearm sEMG [26]. A fully fabric-based glove [27] was developed by Yap’s team to assist in bidirectional finger acting through a flexion actuator and an extension actuator that was integrated into the glove. For this soft glove, four control strategies were offered for rehabilitation training, including passive button driving, cyclic movement training, sEMG intention detection (the desire to activate, hold, or release), and bilateral training through a commercial data glove. However, one of the biggest challenges on sEMG involved hand rehabilitation is that the academic achievements were barely implemented in a commercial system [28]. The important criteria for patients’ widespread acceptance included low consumption, limited complexity, real-time control, minimal training, and so on [28].

In conclusion, although much progress has been made, sEMG-driven soft exoskeletons should be redesigned to drive more subtle hand motions, not just detecting the intent to open or close. Thus, the structure of actuators should be optimized to generate and support more human-like bending states. In addition, the accuracy of subtle hand motion recognition model via sEMG signals should be further improved.

In this paper, we developed a soft exoskeleton glove (SExoG) for hand bilateral training, which can be used to train injured fingers of wearers to complete four types of hand motion: Extension, rest, spherical grip, and fist. When the motion type of the healthy hand is recognized from the sEMG signals of the healthy forearm, The SExoG will drive the disabled hand to perform the same hand movement. The main idea behind the SExoG is to combine two-step algorithms for hand motion recognition from sEMG signals. The first step is sEMG classification by a neural network, the second step is optimization by the state exclusion algorithm.

## 2. Materials and Methods

### 2.1. The Framework of the SExoG System

**Figure 1:**
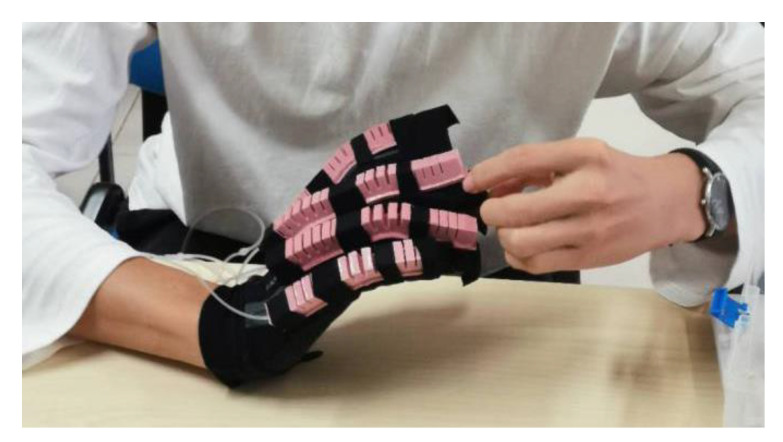
The design of the soft exoskeleton glove (SExoG).

**Figure 2:**
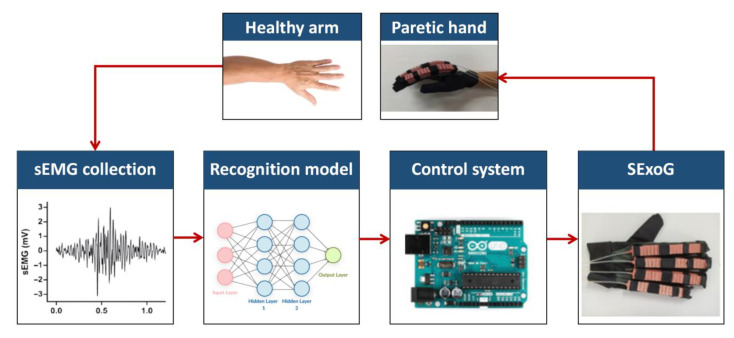
System diagram of the SExoG.

The design of the SExoG system is shown in Figure 1. The SExoG system contains four blocks, including sEMG collection, recognition model, control system, and SExoG. Figure 2 shows the schematic diagram of the SExoG. Firstly, the sEMG signals were collected from the healthy forearm. The recognition model of hand motion intention via sEMG signals was constructed combining neural networks and a state exclusion algorithm. Then the sEMG signals for validation would be fed into the model to generate control messages to drive the robot terminal based on a control strategy. The experiments were tested offline.

**Figure 3:**
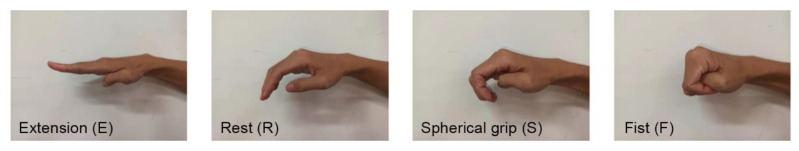
Four hand motions.

The SExoG system can be used to recognize and assist in four hand motions for bilateral rehabilitation training. The four motions are the basic ones in our daily life, as shown in Figure 3. For description, we encoded the names of the motions into four letters: E for Extension, R for Rest, S for Spherical Grip, and F for Fist.

### 2.2. Signal Acquisition

An OpenBCI (Cyton with Bluetooth receiver) board was used for sEMG collection in the experiments. It is an open-source device, which could be furtherly developed to be used for real-time monitoring. Through the OpenBCI GUI, the raw signals were sampled at 250 Hz and band-pass filtered at 5–50 Hz with a notch filter implemented to remove the 50 Hz line interference.

**Figure 4:**
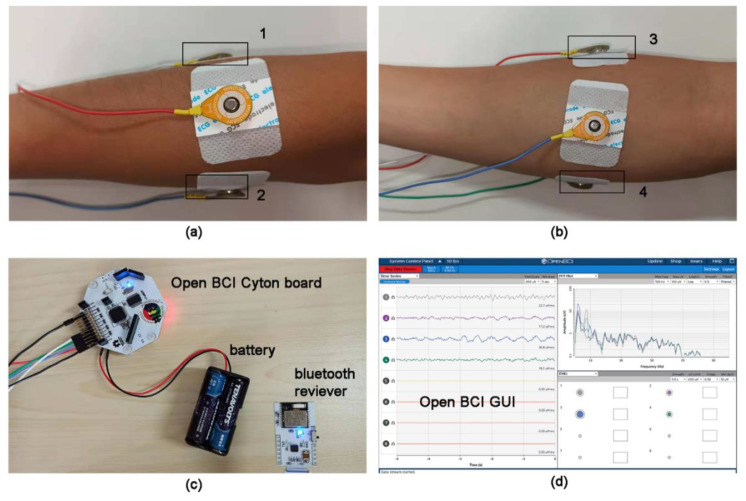
The process of surface electromyography (sEMG) collection. (**a**) The electrode positions of 1 and 2. (**b**) The electrode positions of 3 and 4. (**c**) The hardware for sEMG collection. (**d**) The software of sEMG collection.

Figure 4 shows the process of sEMG collection. The single disposable Ag/AgCl strip electrodes (JK-1, Jun Kang Medical Supplies Ltd., Shanghai, China) were used to record sEMG activities. The electrodes were 5 cm in length and 3.5 cm in width and were pasted on the skin of four positions on the subject’s forearm. A common electrode was used for each channel.

Each of these positions does not correspond exactly to one muscle but is distributed around the arm at a certain distance from the wrist so that these electrodes can form a ring. The reason for this is to position the electrode in an easier and faster way, not only when carrying out repeating experiments but also when applying the SExoG in a real situation. 

The dataset of sEMG signals was randomly divided into two subsets, a training set and test set. The training set was used to construct a machine learning model that maps the sEMG to four hand motions, and the signals were independently stored based on a single motion. The test set was used to validate the SExoG through continuous control messages from sEMG of motion sequences. The details for sEMG acquisition are described in Experiment 2.

### 2.3. The Hybrid Recognition Model

#### 2.3.1. Machine Learning

To generate reliable control messages for the SExoG, a hybrid classification model was designed to recognize the sEMG and exclude impossible results. The hybrid model consisted of two sub-models, including the machine learning method and the state exclusion algorithm. The solution pattern was to classify the sEMG by a neural network and then use the state exclusion algorithm to filter and revise.

First, a neural network was utilized to build the relationship model between sEMG and motions. The root mean square (RMS), as the features of sEMG, were extracted under a frequency of 125 Hz. Though there are many features to choose from, still, RMS has proved its productiveness in many concerning studies. The analysis windows have a duration of 500 ms for feature extraction, and the successive analysis windows are adjacent and disjoint [29]. In this study, the sampling frequency is 250 Hz, which means that two RMS values can be calculated for sEMG signals per second. RMS is calculated for each of the 4 sEMG channels separately. The calculating formula for RMS is:

$$RMS=\sqrt{\frac{1}{N}\sum_{i=1}^{N}v_{i}^{2}},$$

where N is the number of sample points (*N* = 125 samples), and $v_{i}$ is the voltage value at the time i.

**Figure 5:**
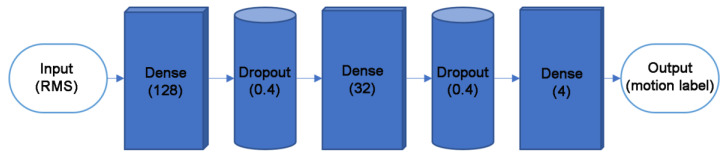
The structure of the neural network.

Now a recognition model could be trained. Given that the discrete signals, the recognition algorithm, has been developed well, many platforms are offering convenient ways to construct neural network models. Among them, Keras, a powerful deep learning frame for Python, was employed to build a neural network in this study. We divided the dataset of single motions into 70% and 30% for model training, including cross-validation, and accuracy testing. Figure 5 shows the structure of the neural network. We tried different configurations of hyper-parameters to train the neural networks repeatedly, and judged the performance of each model by the accuracy curve and loss curve, and then found the appropriate hyper-parameters.

In this neural network, the RMS values of 4 channels were used as the input vectors. Two hidden layers with 128 and 32 nodes were used, and the activation function was “relu”. The output dimension is 4 (E, R, S, and F). The output layer took different activation functions, using “softmax”. The Dense is used to specify the fully connected layer. We set the dropout as 0.4. The learning rate was set as 0.01. We selected “categorical crossentropy” as the loss function. The momentum was set as 0.000001, the decay was set as 0.9, and the batch size was set as 16. The Stochastic Gradient Descent (SGD) was selected as the optimizer.

#### 2.3.2. State Exclusion

Then, the predict results from the machine learning model were transformed into the states of motions, and a conditional state exclusion algorithm was induced to filter impossible situations following each motion. Based on the relationship between the four motions (Figure 3), some of the motion sequences that occur adjacently are impossible, such as RF, SE, FR, FE, EF, and ES, which should be excluded. Table 1 shows the possible motion sequences of the current one and the next one. The motion groups without state transition are RR, EE, SS, and FF. The motion groups without state transition are RE, RS, SR, SF, FS, and ER.

The pattern for this algorithm was to take different actions by the situation. When the current state comfort to any occasion in the above table, it stayed the same way. In other cases, the signal would be replaced by the earlier one to maintain state.

### 2.4. Control Strategy

The control messages in our experiments were directly sent from Python, received and conducted by Arduino (Arduino Uno, Arduino) through serial communication. The Python run on a computer with a 3.40 GHz Intel Core i5-7500 CPU and 8 G RAM. The strategy in this process was to wait for one motion to be completed before sending the next one, so the timing was not taken into consideration. Under the circumstances, we focused on whether the system can carry out the sequences with assigned motions. 

**Figure 6:**
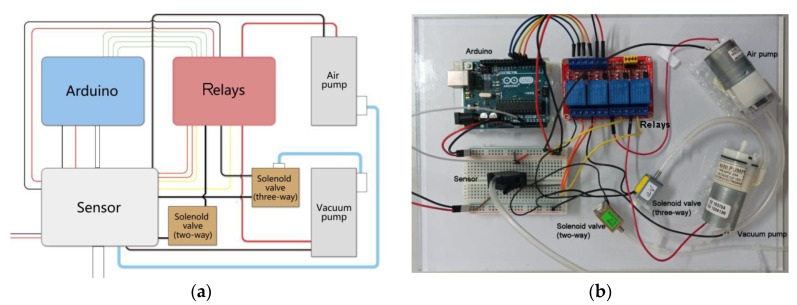
The control board. (**a**) The schematic diagram. (**b**) The prototype.

The electro-pneumatic system mainly consisted of a microcontroller, an air-pump (MAP-AM-265, Mitsumi, Tokyo, Japan), a vacuum-pump (DZ 15370A, Beyok, Dongguan, China), a pressure sensor (MPX5100, NXP, Eindhoven, The Netherlands), miniature solenoid valves, relays (JQC-3FF-S-Z, Tongling, China), and an adapter. We integrated these elements on an acrylic board as shown in Figure 6. The size of the board is 300 × 200 × 8 mm, which is migrated-friendly.

**Figure 7:**
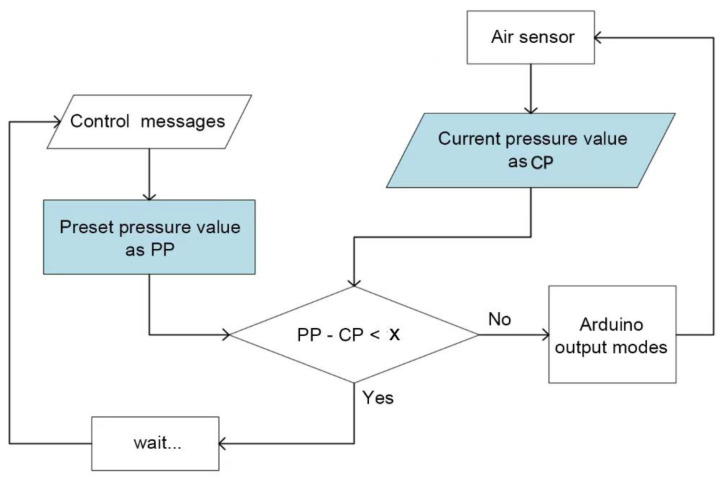
Control strategy.

The microcontroller Arduino could shift between output modes of either supply air, release air, extract vacuum, or stay loop close. According to Figure 7, the control messages were compared with real-time states to decide the power mode. The control messages were transformed into the corresponding preset pressure that was able to assist in specific hand motion. The current pressure was monitored upon an air sensor. If the value of PP were close to that of CP, the system would delay and wait for the next control message; or the control system would keep adjusting until meeting the condition. When PP-CP is less than an acceptable value of error x. When x is infinitely close to 0, CP is much closer to PP. However, in this experiment, taking into account the performance of air pump, we set x as 1 KPa for RE, and we set x as 5 KPa for SF and RS.

### 2.5. Actuator and Prototype

#### 2.5.1. Design

**Figure 8:**
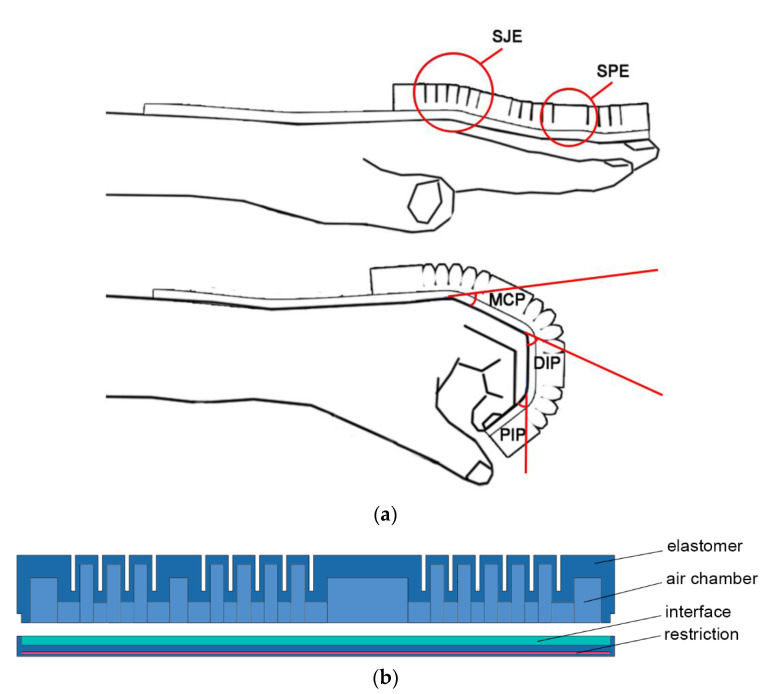
The design for the actuator. (**a**) The design reason for the actuator. (**b**) The detail of the structure for the actuator.

Since the only bending part of the fingers are the joints while the phalanges remain straight, the actuators were designed complying with this regulation. As shown in Figure 8a, the soft joint exoskeleton (SJE) and the soft phalange exoskeleton (SPE) were embodied in an actuator. The SJEs cover the finger joint, including the metacarpophalangeal (MCP), distal interphalangeal (DIP), and proximal interphalangeal (PIP) while the SPEs cover the phalange.

Figure 8b shows the structure of the actuator. There were some disparities between the chambers of the SJE and SPE. For the SJEs, several narrow chambers were rowed to contribute to the finger’s bending. The chambers were smaller in length, and a couple of chambers were sequenced to function. Also, the sidewalls were thin so that they would easily squeeze on each other under air pressure. However, the chambers from SPE, designed for maintaining, were within a certain length according to the length of the different phalange. Its top walls were thicker than that of the SJE to restrain the radial deformation.

We designed both the elastomer surface and air chamber with variable stiffness. According to the skeletal characteristics of the human hand, the elastomer bends at the joints, but it should be straight at the segments. We designed a structure with variable stiffness at different localities of the elastomer, which is different from the work of Polygerinos [4]. We also designed the air chamber with variable stiffness to replace the pneumatic channels inside the actuator in [24], as shown in Figure 8b. We also added an interface to reinforce the bottom, so as to prevent air leakage between the bottom of the elastomer and restriction layer made of non-woven fabric. The reinforced bottom of the actuator can improve the success rate of molding effectively.

Then, two layers were added to the bottom to seal the chambers: An elastomer one as the base and a piece of non-woven fabric embedded inside. The non-woven layer can constrain deformation along the bottom of the actuator. So when the air was pumped in, the actuator curves in the shape of a finger because of the impact from the chambers.

**Figure 9:**
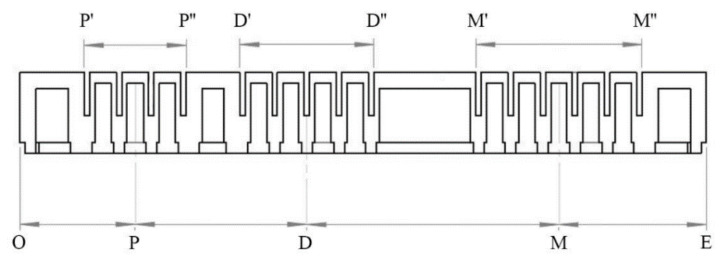
The customization scheme of actuator size.

Besides, the geometrical parameters of the actuators were customized for the subject. We proposed a customization scheme of actuator, as shown in Figure 9. Taking the index finger for example, the geometrical parameters of the actuators were derived as follows:

Step 1: Cut A4 paper into a strip with the width of 1 cm and place it on the surface of the finger to be measured.

Step 2: Align one end of the strip with the tip of the index finger and use the tip as the origin O. From this point, visually locate the finger’s position corresponding to the center of PIP, DIP, and MCP. Use points P, D, and M, respectively, and mark on the strip with a pencil.

Step 3: Start from point P, Point D, and point M, respectively, find the starting and ending positions of the spanning joint length on the left and right sides of this point. Accordingly, use points P′ and P”, D′ and D”, and M′ and M” respectively, and mark them on the paper with a pencil.

Step 4: Start from point M, find a suitable point E in the wrist direction so that OE is the total length of the actuator, and point E is to the right of point M”, and then mark them on the paper with a pencil. At this point, the size customization of the actuator corresponding to the index finger is completed.

In this paper, we used the average values across the four subjects. The actuators, at the length of 133 mm, 128 mm, and 108 mm, were respectively designed for the middle finger, the index as well as the ring fingers, and the little finger.

#### 2.5.2. Fabrication

The actuators were manufactured via the followed steps: (i) Mix up the silicone A and B (Elastosil M4601, Wacker, Munich, Germany) by the ratio of 9:1; (ii) pour the silicone into the main mold; (iii) put the 3D printed molds (Weilai 8500, 3D, China) into a vacuum chamber to de-gas on the silicone; (iv) pop the bubbles on the surface and remove the extra silicones around the edge; (v) place the mold in a heat oven at 60 C and wait for 30 min; (vi) pour a layer amount of silicone into the base mold, and add an non-woven layer on the top; (vii) take the top piece of the mold off and pull the elastomer apart from the bottom mold; (viii) assemble the elastomer with the base by one more layer of silicone; (ix) heat the assembled actuator for another 30 min and take it off from the base mold.

After the four actuators were fabricated, they were attached to a glove by sewn cloth loops. The glove would be the executor in the SExoG.

### 2.6. Experiment 1: The Acquisition of Pressure Values

This study was approved by the Ethics Committee of East China University of Science and Technology. Four healthy male subjects (mean ± SD, age = 24.5 ± 0.58 years, hand length = 188.75 ± 2.98 mm) volunteered to participate in the experiments. Before the experiments, the age and hand length of subjects were recorded. The purpose of experiment 1 is to acquire the corresponding pressure values of air-pump to generate corresponding angels of the whole SExoG to support four hand motions.

#### 2.6.1. The Acquisition of Pressure Values for the Unloaded Actuators

We need to find the specified values of air pressure to perform the four hand motions of the actuators. The unloaded (not wore in hand) actuators was tested under different air pressure, that of −2, 0, 10, 20, 30, 40, 50, and 60 KPa. Each actuator was photographed.

**Figure 10:**
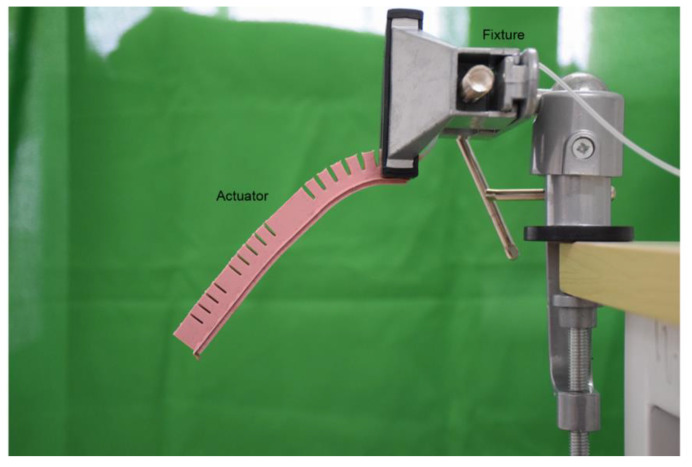
The experimental scenario.

As shown in Figure 10, a fixture was used to clamp the end of the actuator to imitate the real flexing of a finger. In this case, the restricted end corresponds to the metacarpophalangeal joints that connect the metacarpal bone and the finger bone.

The bottom contour lines were drawn out from the photos to indicate the bending status. The contour lines were listed together to show actuator expanding as the pressure growing. Also, we measured the three angles (MCP, PIP, and DIP) of each SJE regarding the purposive design of the actuator.

The test process was conducted via the Arduino with an air sensor monitoring the pressure inside the actuator. To test the bending angles of the actuator for the rest, spherical grip and fist, an air pump was used to feed air into the actuator. Once the air pressure reached a preset value, the pump was closed, and the shape of the actuator was photographed. For the extension, the air was extracted out by a vacuum pump, and the shape of the actuator was also photographed.

#### 2.6.2. The Acquisition of Pressure Values for the SExoG Worn on Human Subjects

The pressure values for driving the whole SExoG worn on human subjects should be determined. The subjects were required to be relaxed, so that the exoskeleton could be driven by air with less resistance.

The purpose was to find out the pressure values for the SExoG to assist in the four hand motions respectively. For motion E, the control system was set as air extracting mode. For R, the system was inactive. For S and F, the control system was set as air supply mode and stop at the satisfied states. The shape states for each motion as well as the corresponding pressure values were recorded.

### 2.7. Experiment 2: The Acquisition of sEMG Signals

The experimental protocols were introduced and signed by the subjects with acknowledge. Besides, the subjects were informed to wear a sleeveless shirt and the skin surface on the tested arm was cleaned before attaching electrodes.

For the first stage, the labeled sEMG activities for a single motion was required. The subject was asked to conduct and maintain the four motions of the hand, including extension, rest, spherical grip, and fist. We separated the process into short-time sections in order to avoid muscle fatigue. For each independent experiment, the subject offered continuous valid signals for about 20 s and would then take a rest. After 30 times repeating, we collected 10 min duration of sEMG in summary for each hand motion. Based on this way, the sEMG for other hand motions were obtained. It took four days to complete all the experiments for one subject. The electrodes were taken off re-placed among the days, which might enhance the generalization of the signal classification model.

**Figure 11:**
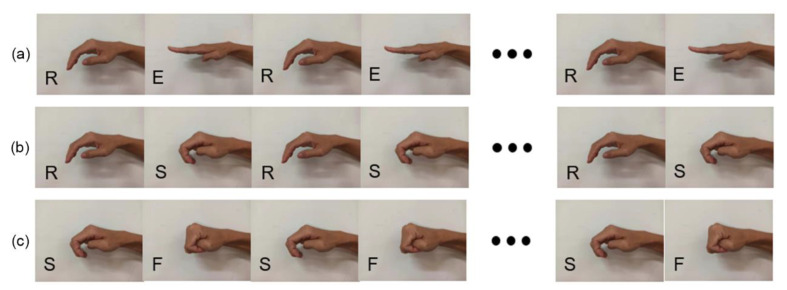
The designed hand motions sequences. (**a**) The motion sequence RE. (**b**) The motion sequence RS. (**c**) The motion sequence SF.

For the second stage, the sEMG was registered without precise labels because the switching time points between hand motions were not explicit. The subject was requested to conduct the motion sequences (Figure 11) by switching between two motions every five seconds and repeated for five times.

### 2.8. Experiment 3: The Performance Evaluation of the SExoG System

The objective of Experiment 3 is to evaluate whether the SExoG system can mirror the motion sequence of non-paretic hand precisely. The sEMG signals and repeat motions were both measured. The SExoG was set to repeat motions of a designed sequence to detect whether all the motions can be conducted in right order. Every two adjacent actions form a loop. The designed sequences were shown in Figure 11, covering all possible adjacent motion situations.

When the SExoG was driven by the control messages, the air pressures were traced by the sensor simultaneously. The corresponding time points were also recorded as the time dimension. The system performance was determined by the number of motion loops recognized from the whole motion sequence.

## 3. Results

### 3.1. The Results of Pressure Values of Unloaded Actuators

**Figure 12:**
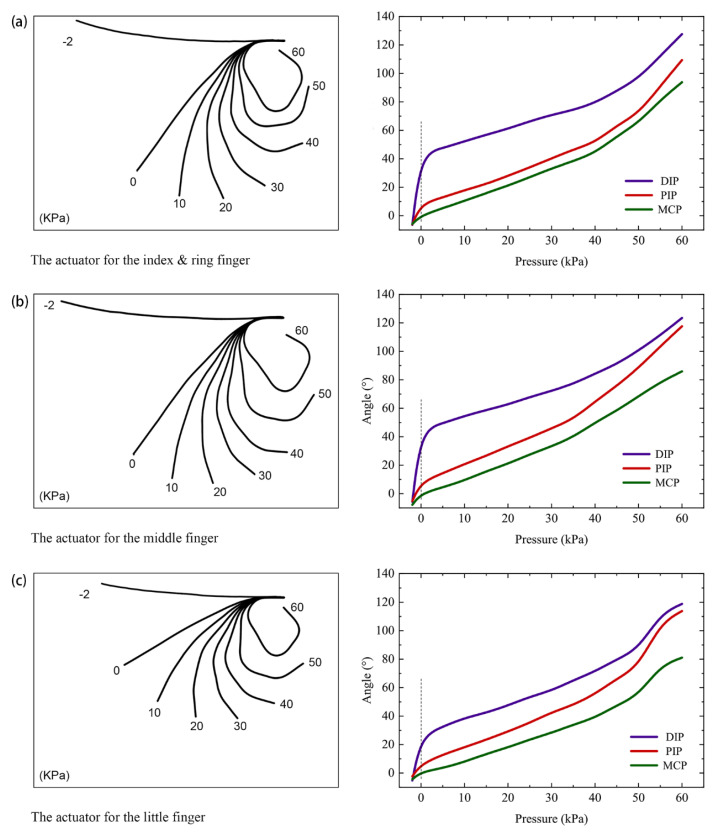
Actuator performance under different air pressure. (**a**) The results for the actuator of 128 mm length. (**b**) The results for the actuator of 133 mm length. (**c**) The results for the actuator of 108 mm length.

The photos of the actuators under different pressure were imported into CAXA CAD 2015 (The CAXA Inc., Beijing, China), and then the bottom contour lines were traced out from the photos. Finally, the corresponding angle values of MCP, PIP, and DIP were measured in CAXA. Figure 12 shows the changing shapes of each actuator by putting the contour lines together, as well as the three angles of SJEs, as air pressure growing from −2 to 60 KPa. As shown in Figure 12, the actuators comply with the finger in the shape of flexion or extension. With the restriction of a fixture, a nature hanging state of an actuator was presented. When the air was pumped in, the actuator gradually flexed on the SJE position. The angle values of MCP, PIP, and DIP are also shown in the figure.

Compared with the active range of motion (ROM) for each finger [30] (Table 2), the angles from the unloaded actuators seem to be beyond the active range. However, when the actuators were assembled into the glove, there were additional limitations on the bending performance. Also, when one subject wears the glove, there would be the reaction force from the hand skeleton. So, in a real-life scenario, these angles from the SExoG will not go beyond the reasonable range.

### 3.2. The Results of Pressure Values of SExoG Worn on Human Subjects

**Figure 13:**
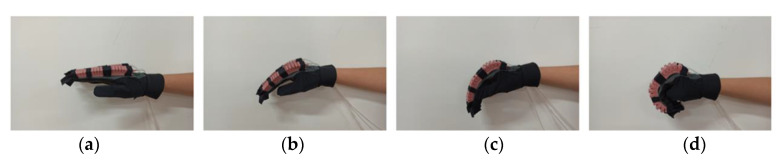
The four wearing states of sExoG. (**a**) Extension, (**b**) Rest, (**c**) Spherical grip, (**d**) Fist.

Figure 13 shows the four states of one selected subject wearing the SExoG. The pressure at −2 Kpa can yield the motion of extension, the pressure at 0 Kpa can yield the motion of rest, the pressure at 40 Kpa can yield the motion of spherical grip, and the pressure at 70 Kpa can yield the motion of fist. The pressure values for the four human subjects are the same.

From the wearable ability evaluation, the subjects who wore the exoskeleton showed no discomfort with wearing the device. The exoskeleton performed the four motions of hand for the subject. The pressure values that contributed to the motion of E, R, S, and F were approximately at −2, 0, 40, and 70 KPa. This result illustrates that when the exoskeleton was loaded, larger pressure would be required to reach the shape like independent actuators.

### 3.3. Recognition Results of Four Motions from sEMG Signals

The classification accuracy of the neural network model was judged by the discrete test signal. After the neural network was well trained on the labeled sEMG, the new RMS data were fed into the model to get classification results. The results were compared with the true hand motion labels to calculate accuracies (Table 3).

Concerning the sequence signals generated from continuous motions, firstly, they were fed into the neural network model for prediction. The results were transformed and presented in the four motion labels, and then filtered by the exclusion of the state (Table 4). After that, the signals were used as control messages.

**Figure 14:**
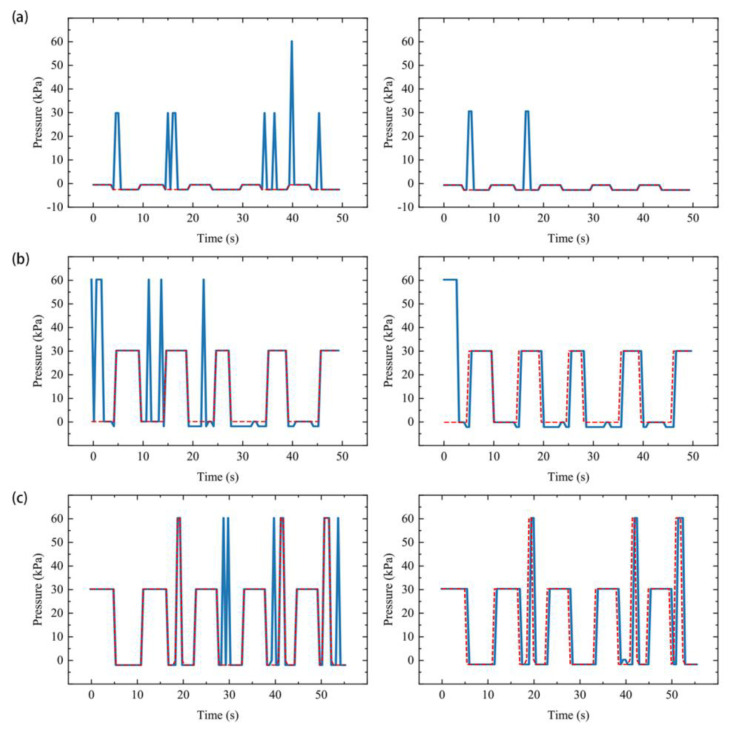
The pressure values transformed from the model prediction of the three motion sequences. The left graphs show the results after the neural network model (blue lines); the right graphs show the results after the state exclusion (blue lines). The red lines show the ground truth pressure values. (**a**) The results for motion sequence RE. (**b**) The results for motion sequence RS. (**c**) The results for motion sequence SF.

For visualization, we transformed the classification results E, R, S, and F to the preset pressure −2, 0, 30, and 60 KPa, respectively, based on the actuator performance. Then the continuous signals were visualized, as shown in Figure 14. In other words, Figure 14 shows the ideal pressure graph from the system when driven by control messages of the specific motion sequences. 

### 3.4. SExoG Performance

**Figure 15:**
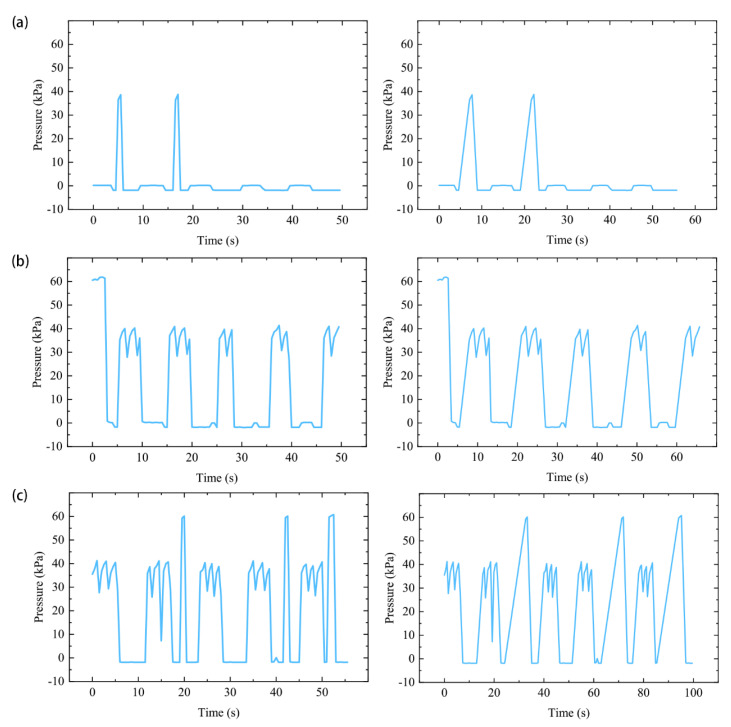
Actual air pressure among the motion sequences. The left graphs show the results of that when the time is out of consideration; the right graphs show the results of that when time is taken into account. (**a**) The results for motion sequence RE. (**b**) The results for motion sequence RS. (**c**) The results for motion sequence SF.

The air pressures of each motion sequence were plotted under time dimension, as shown in Figure 15. Ideally, 10 motions should be detected for each motion sequence according to the signals acquiring process. There are 10 motions in total, repeated by two motions or each motion sequence.

Without considering the hardware response time, the trend of the ground truth pressure curve and the target pressure curve after the state exclusion is consistent (Figure 14 and Figure 15), which means that the SExoG can perform the action orderly according to the control message. However, in the process of actual execution, it is necessary to inflate and pressurize the actuator to achieve different bending states. The aeration time increases with the increase of the target pressure value, so there will be a certain delay. However, the effect caused by this kind of delay can be weakened by the improvement of hardware performance, or even be basically eliminated. In summary, continuous sEMG was delivered to the hand exoskeleton in an orderly manner, demonstrating the effectiveness of the SExoG system.

## 4. Discussion

In this work, we developed a low-cost soft exoskeleton glove (SExoG) system driven by sEMG signals from non-paretic hand for bilateral training. The novel SExoG system can mirror the hand motions of non-paretic hand with good performance across four hand motions (extension, rest, spherical grip and fist).

The mean accuracy for concrete sEMG classification is 96.6% (Table 3). The accuracy can well support the afterward state exclusion. The SExoG could be improved if the concrete signal recognition was better developed. Also, as shown in Figure 14, unreasonable situations were effectively filtered through the state exclusion, and the mean accuracy was further improved to 98.7%. The recognition performance attained with our hybrid algorithm is better than that achieved by traditional ones [16,17,18]. When these impossible states are excluded, the conditions of dramatic state change will not occur, such as a straight jump from −2 KPa to 70 KPa, or a plunge from 70 KPa to −2 KPa. The processed signals were then transmitted as control messages. Thus, the control system could remain more stable when receiving these messages.

The system can conduct the motions based on the supervisor’s control messages successfully. First of all, we can find that the actual pressure-time graphs are consistent with the ideal one by comparing Figure 15 to Figure 14. As shown in Figure 15, for the RE sequence, 5 of the repeated scenarios can be recognized with three perfect times; for the RS sequence, four times are well presented; for the SF sequence, three times could be recognized. In a word, the continuous sEMG was orderly transmitted to the actuators to prove the function of the SExoG. 

In this study, we only collected the sEMG signals of forearm muscle groups, but the recognition results are encouraging. Although the hand bilateral training involves both the finger and wrist movements, surface EMGs can be recorded from intrinsic hand and forearm muscles that produce these movements. However, the functional recovery movements may arise electrode shift of hand, which will interfere the collection of EMG signals from hand and reduce the recognition accuracy [29]. In addition, previous studies have shown that the recognition rate of forearm EMGs is higher than that of hand EMGs [31]. Therefore, forearm muscles can be leading muscles for investing the SExoG system in further study.

Overall, our findings are more optimistic than those of Polygerinos et al. [26], who only detected the intent to open or close via sEMG signals. We used low-cost open source hardware and traditional electrodes to detect more subtle hand motions. In future work, the electrodes with a smaller surface area, more channels, and better signal-to-noise ratio, such as microelectrode arrays [32], can be used for detecting more complicated hand motions, involving much more precise location on specific muscle groups. 

However, some limitations still need to be further improved:

(1) The dataset in our experiments was restricted to only four subjects. An enlarged dataset is required for clinic devices developing after the prior study. Also, more motion classes would be better for hand exercise. It appears that the ground truth for each hand motion type is personalized to each subject. Individual calibration might also be expected for each subject, when the exoskeleton is put into real-life use.
(2) For the four hand motions in this study, the motion state change of the thumb is small, so the thumb actuators was not fabricated. When it comes to more subtle hand movements, the thumb should be taken into account in future study.
(3) Because of the pump capacity, it took more time than that of the raw motion routine for the actuator to reach certain states. In other words, there was a delay when following continuous control messages. The system would have a better real-time reflection within a delay duration under 300 ms.
(4) There are four tubes to supply air for the pneumatic actuators of the exoskeleton. These tubes might cause clutter or inconvenience for the subject to wear and rely on. Before the exoskeleton put into the clinic application, the air tubes connection would be optimized via integration.

## 5. Conclusions

In this paper, we promoted a SExoG for bilateral hand rehabilitation. The system was evaluated on both concrete sEMG recognition and continuous motion transform. We built the system at low consumption with open accessed software and hardware. The actuators of the soft exoskeleton were designed to comply with the finger skeleton structure. The exoskeleton was tested by healthy subjects to confirm comfort and safety. In the future, the clinic tests should be involved in more valuable evaluations. 

## Figures and Tables

**Table 1:** The motion sequences of the current one and the next one.

Current Motion	Next Motion	The Motion Groups with State Transition
R	R, E, S	RE, RS
S	S, R, F	SR, SF
F	F, S	FS
E	E, R	ER

**Table 2:** Mean range of motion (ROM) for finger joints.

Joint	Extension (°) (Mean ± SD)	Flexion (°) (Mean ± SD)
DIP	−6.2 ± 4.2	84.5 ± 8.3
PIP	−7.6 ± 3.5	101.3 ± 8.1
MCP	−19.3 ± 6.4	90.4 ± 9.2

**Table 3:** The average accuracy of the neural network model across four subjects.

Hand Motion Type	Model Accuracy (%)	Standard Deviation
E	92.61	0.67
R	98.47	1.72
S	97.22	0.61
F	98.11	0.48
Mean	96.6	0.51

**Table 4:** The average accuracy after optimization by the state exclusion algorithm.

Hand Motion Type	Model Accuracy (%)	Standard Deviation
E	97.06	0.68
R	99.45	1.68
S	98.98	0.63
F	99.31	0.45
Mean	98.7	0.53

## Data Availability

The data presented in this study are available on request from the corresponding author.
